# Screening for Potential Compounds Using Drug-Repurposing of N-Methyl-D-Aspartate (NMDA) Receptor for Autism Spectrum Disorder (ASD)

**DOI:** 10.21315/tlsr2025.36.1.12

**Published:** 2025-03-30

**Authors:** Nordina Syamira Mahamad Shabudin, Ahmad Naqib Shuid

**Affiliations:** 1 Department of Biomedical Sciences, Advanced Medical and Dental Institute, Universiti Sains Malaysia, 13200 Bertam, Kepala Batas, Pulau Pinang, Malaysia; 2 Department of Community Health, Advanced Medical and Dental Institute, Universiti Sains Malaysia, 13200 Bertam, Kepala Batas, Pulau Pinang, Malaysia

**Keywords:** Autism Spectrum Disorders (ASD), N-methyl-D-aspartate (NMDA) Receptor, Docking, Molecular Dynamics Simulation, ADMET Studies, Sindrom Spektrum Autistik (SSA), N-methyl-D-aspartate (NMDA) Reseptor, Saringan Maya, Simulasi Dinamik, Kajian ADMET

## Abstract

In Malaysia, the study on autism spectrum disorders (ASD) is limited. Most studies only focus on gene neuroligin 3 (NLGN3), NLGN4X, neurexin 1 (NRXN1) and SH3. This study focuses on the N-methyl-D-aspartate (NMDA) that was believed to have a significant effect on ASD. In this study, potential compounds and drugs that can restore receptor function in autistic patients were analysed. This research used an effective in silico method known as drug-repurposing to discover and rediscover drugs and analyse the binding of potential compounds or drugs to the NMDA receptor. AMPA and DOCK4 were used as controls in this study. Using a trusted server, Drug ReposER, 13 potential compounds or drugs that bind to NMDAR were identified. Then, proceed to the docking of potential compounds or drugs that bind to the NMDA receptor using Autodock Vina, Autodock, Hdock and CB dock and three drugs were selected that have the best binding score to NMDA, AMPA and DOCK4. The drugs were alitretinoin, salicylic acid and indinavir, respectively. Next, molecular dynamics simulations were performed with all selected compounds to study drug-protein binding, with detailed analysis of bond stability using root-mean-square fluctuation (RMSF) oscillations. Finally, ADMET (Absorption, Distribution, Metabolism, Excretion and Toxicity) predictions identify 4-androstenedione, tryptophan, carbocisteine and vitamin A as having minimal toxic effects. This study showed that alitretinoin, which was known to treat skin lesions from Kaposi’s sarcoma, might have the ability to reverse the effect in ASD, particularly in NMDA receptors, potentially making a significant impact on the field of neurology and psychiatry.

## Abstract (fr)

**Abstrak:**

Di Malaysia, kajian mengenai Sindrom Spektrum Autistik (SSA) amat terhad. Kebanyakan kajian hanya memberi tumpuan kepada gen neuroligin 3 (NLGN3), NLGN4X, neurexin 1 (NRXN1) dan SH3. Kajian ini memberi tumpuan kepada N-methyl-D-aspartate (NMDA) yang dipercayai mempunyai kesan yang signifikan terhadap SSA. Dalam kajian ini, beberapa sebatian dan ubat yang berpotensi untuk memulihkan fungsi reseptor dalam pesakit autisme telah dianalisis. Penyelidikan ini menggunakan kaedah *in silico* yang berkesan yang dikenali sebagai *drug-repurposing* dan saringan maya untuk menemui dan menemui semula ubat serta menganalisis pengikatan sebatian atau ubat berpotensi kepada reseptor NMDA. AMPA dan DOCK4 digunakan sebagai kawalan dalam kajian ini. Menggunakan servis laman sesawang yang dipercayai, Drug ReposER, 13 sebatian atau ubat yang berpotensi berinteraksi dengan NMDAR telah dikenal pasti. Seterusnya, saringan maya bagi sebatian atau ubat berpotensi yang berinteraksi dengan reseptor NMDA dilakukan menggunakan Autodock Vina, Autodock, Hdock dan CB dock, dan tiga ubat dipilih yang mempunyai skor interaksi terbaik kepada NMDA, AMPA dan DOCK4. Ubat-ubatan tersebut ialah alitretinoin, asid salisilik dan indinavir. Seterusnya, simulasi dinamik molekul telah dijalankan dengan semua sebatian yang dipilih untuk mengkaji pengikatan ubat-protein, dengan analisis terperinci kestabilan ikatan menggunakan graf RMSF. Akhirnya, ramalan ADMET (Penyerapan, Pengedaran, Metabolisme, Perkumuhan dan Ketoksikan) mengenal pasti 4-androstenedione, tryptophan, carbocisteine dan vitamin A mempunyai kesan toksik yang minimum. Kajian ini menunjukkan bahawa alitretinoin, yang diketahui merawat lesi kulit daripada sarkoma Kaposi, mungkin mempunyai keupayaan untuk membalikkan kesan dalam SSA kepada normal, khususnya dalam reseptor NMDA, yang berpotensi memberi impak yang ketara dalam bidang neurologi dan psikiatri.

**Highlights**

- The study used advanced tools like Drug ReposER, Autodock Vina, Autodock, Hdock and CB dock to find drugs that bind effectively to the NMDA receptor.
- Thirteen drugs were identified to improve synaptic communication. Alitretinoin, salicylic acid and indinavir showed the strongest binding to key proteins (NMDA, AMPA and DOCK4).
- Simulations and safety predictions (ADMET) showed that some drugs, like 4-androstenedione and tryptophan, are stable and have minimal side effects, making them suitable for further research.

## INTRODUCTION

Autism, a spectrum disorder with a range of severity, spanning from moderate to severe, is a pressing issue in Malaysia due to the limited research and knowledge on autism spectrum disorders (ASD) (Lord *et al*. 2018). The Malaysian public has moderate knowledge (72%) and a low positive attitude (57%) toward autistic individuals and families (Che Azmi *et al*. 2022). In 2021, 589 children aged 18 and below were diagnosed with ASD, a 5% increase from 562 children in 2020. This substantial rise from just 99 children diagnosed in 2010 underscores the urgency of comprehensive epidemiological studies on ASD in Malaysia. The lack of such studies complicates the development of effective support systems and interventions for affected families (Eow *et al*. 2020).

Due to the heterogeneous nature of autism as a spectrum condition, the efficacy of specific treatments may vary among individuals. According to the study conducted by Coleman *et al*. (2019), neuroscience and genetics have identified intriguing risk patterns, albeit with limited practical utility. Further investigation is necessary to ascertain the efficacy of cognitive and psychiatric therapies for various populations of children, particularly those with significant comorbidities. According to Lord *et al*. (2018), given the widespread comorbidity of ASD with other disorders, it is common for individuals with ASD, both children and adults, to be prescribed psychiatric medications (Coleman *et al*. 2019). According to Madden *et al*. (2017), these drugs effectively mitigate frequently observed symptoms and maladaptive behaviours such as aggression, depression, insomnia, impaired concentration, tantrums and seizures. Psychiatric medication is frequently given to those with ASD. However, it is noteworthy that the U.S. Food and Drug Administration (FDA) has only approved aripiprazole and risperidone for this specific reason. These medications have effectively reduced irritability in individuals with ASD, as supported by studies conducted by Fusar-Poli *et al*. (2019) and LeClerc and Easley (2015).

While atypical antipsychotic medicines can effectively address behavioural issues in ASD, their long-term usage raises significant concerns. Correll (2009) suggests that adverse effects on glucose and lipid metabolism and body weight can become apparent within 12 weeks. A study by Bobo *et al*. (2013), revealed a notable increase in the incidence of type 2 diabetes and cardiovascular disease. In 2013, Williams *et al*. found no empirical evidence supporting the efficacy of selective serotonin reuptake inhibitors (SSRIs) despite prior research suggesting potential benefits. The authors of the study also indicated that SSRIs may have adverse effects on individuals with ASD. These findings underscore limitations of current pharmacological interventions and the urgent need for a more comprehensive approach to ASD treatment, as current pharmacological interventions have significant limitations.

Addressing the needs of adult patients with ASD presents a significant challenge due to the absence of definitive guidelines. The medications that have received approval from the FDA are typically administered to paediatric patients. Additionally, the Diagnostic and Statistical Manual of Mental Disorders, Fifth Edition (DSM-5), specifically focuses on the diagnostic criteria that are observable in children rather than adults. The identification of ASD in adults necessitates a considerable amount of time. It relies heavily on the expertise and proficiency of clinicians, as people can conceal the symptoms associated with the disorder. The availability of data on medication use in maturity is restricted.

A considerable body of research has primarily concentrated on pharmacological interventions aimed at ameliorating the symptoms of ASD, akin to a temporary solution. However, the complex nature of ASD necessitates a more comprehensive and proactive approach. This study aims to take a step forward by proposing a novel approach. By using one selected drug with the potential to restore the function of N-methyl-D-aspartate (NMDA) plus 12 more drugs tested against other receptors from the same brain area that have a connection in autism that is α-amino-3-hydroxy-5-methyl-4-isoxazolepropionic acid (AMPA) receptor and dedicator of cytokinesis 4 (DOCK4) (Chanda *et al*. 2016; Guo *et al*. 2021; 2023; Kim *et al*. 2019; Niescier & Lin 2021), we aim to bring innovation and progress to the field of ASD treatment. The docking result then being compared and analysed carefully. To further analyse the drugs, molecular dynamics simulations were conducted to see the interaction between each drug with the receptors. Lastly, ADMET studies were employed to evaluate their efficacy and safety.

Even though in silico studies have given comprehensive and reliable results, experimental validation of the identified compounds’ efficacy and safety in biological models is still needed. This study can guide future studies to better understand the characteristics of the compounds and drugs selected for ASD treatment.

## MATERIALS AND METHOD

### Acquisition and Verification of Proteins Structure

The crystal structures of the NMDA receptor (PDB ID: 7EOS) and the AMPA receptor (PDB ID: 7F3O) were obtained from the Protein Data Bank (PDB).

The DOCK4 (Q8N1I0) FASTA sequence was obtained through the UniProt database (https://www.uniprot.org/). The structural prediction was conducted with AlphaFoldDB.

The validation of the proteins was conducted using the Ramachandran plot and SAVES v6.0 tools, specifically ERRAT and Verify-3D.

### Identification of Potential Drugs Candidate That Bind to the Proteins via Drug-repurposing

The crystal structure has been uploaded to the Drug ReposER server (http://211.25.251.163/drrposer/). To predict amino acid structures that exhibit similarity to established drug-binding surfaces, with the aim of facilitating future drug development and implementation. The medicines with the greatest affinity for known drug-binding surfaces were chosen.

### Molecular Docking using Autodock Vina, Autodock, Hdock and CB dock

#### Autodock Vina and autodock

The protein structures underwent modifications involving the removal of water molecules, the addition of polar hydrogen ions, and the incorporation of Kollman charges. The proteins are subsequently stored in PDBQT files for further processing.

The chosen pharmaceutical compounds underwent a reformulation process involving the introduction of partial charges. The medications were preserved in the form of PDBQT files.

The Autodock Vina was run using the script in Appendix A and Autodock was run using the script in Appendix B.

#### Hdock and CB dock

All the proteins and drugs were submitted to Hdock server (http://hdock.phys.hust.edu.cn/) and CB dock server (http://clab.labshare.cn/cb-dock/php/blinddock.php) to dock again.

### Molecular Dynamic Simulation Using GROMACS

Molecular dynamic (MD) simulation was run using the script in Appendix C.

### ADMET Study using ADMET Predictor^®^

To obtain the ADMET Predictor^®^ programme, you can visit the official website of Simulations Plus Inc. and download the software’s installation package. You are advised to select or create a directory within your system where the files related to the ADMET Predictor^®^ should be stored. The specified location will function as the software installation site. The directory was chosen based on its ability to provide accessibility and convenience for you. In order to install ADMET Predictor^®^, you are required to initially acquire the installation package and subsequently designate a directory for installation. It is advisable to adhere to the installation instructions provided by Simulations Plus Inc. as stated in the installation guide.

The software setup process empowers you to configure the installed programme to align with your needs and preferences. This includes your control over the predictive features, such as providing necessary input files, adjusting model parameters, and choosing preferred predictive features. The unpredictability of configuring ADMET Predictor^®^ necessitates consulting the user handbook or literature provided by Simulations Plus Inc. for comprehensive assistance on the precise procedures.

To utilise the ADMET Predictor^®^, it is essential to undertake the installation and setup processes initially. Subsequently, the software can be used by executing the specified command or initiating it from the directory in which it was installed. The software, utilising machine learning techniques, will accurately predict many characteristics, such as solubility, logP, pKa and specific enzyme binding sites (Kumar *et al*. 2021), giving you confidence in its results.

## RESULTS

### Acquisition and Verification of Protein Structure

This study selected three proteins, namely NMDA, AMPA and DOCK4, for investigation. AMPA and DOCK4 were used as control measures.

The structural integrity of the proteins of interest, obtained from the Protein Data Bank (PDB) and Universal Protein Resource (UniProt), was assessed using the Ramachandran plot, ERRAT and Verify-3D service.

The proteins were obtained from the PDB, with the NMDA receptor having the identifier 7EOS and the AMPA receptor having the identifier 7F3O. On the other hand, the DOCK4 protein was obtained from the UniProt, with the identifier Q8N1I0. The verification of all proteins was conducted by the utilisation of the Ramachandran plot and SAVES tools, specifically ERRAT and Verify-3D.

Next SAVES v6.0 were used to validate the protein. ERRAT and Verify-3D were specifically used. The results are shown in the Table 1.

### Identification of Potential Drugs Candidate That Bind to the Proteins via Drug-repurposing

There were 15 potential drugs identified using Drug ReposER. All the drugs were listed in Table 2.

### Docking Results

For this part, Autodock Vina, Autodock, Hdock and CB dock were used to run the docking and compare each software’s results.

### Molecular Dynamics Simulation Using GROMACS

**Figure 1:**
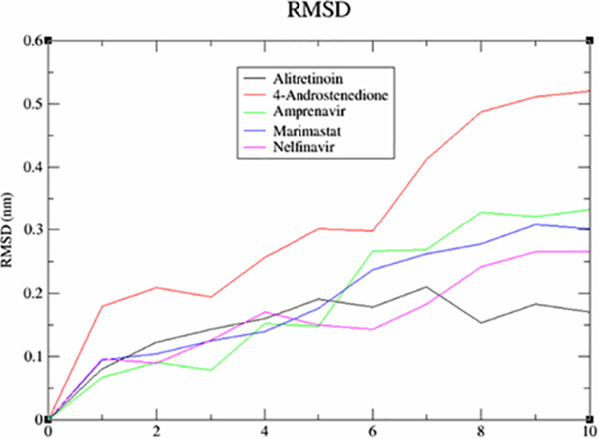
The analysis of RMSD for NMDA.

**Figure 2:**
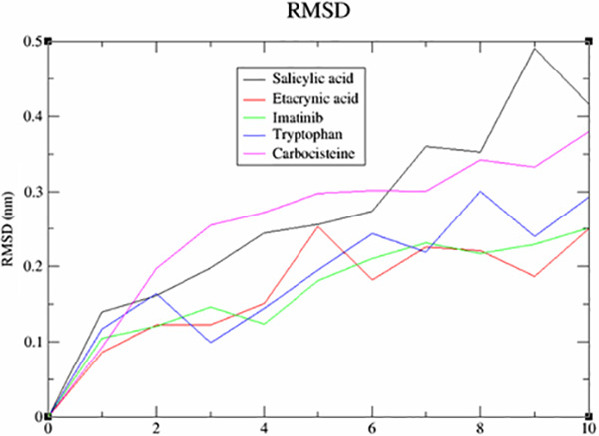
The analysis of RMSD for AMPA.

**Figure 3:**
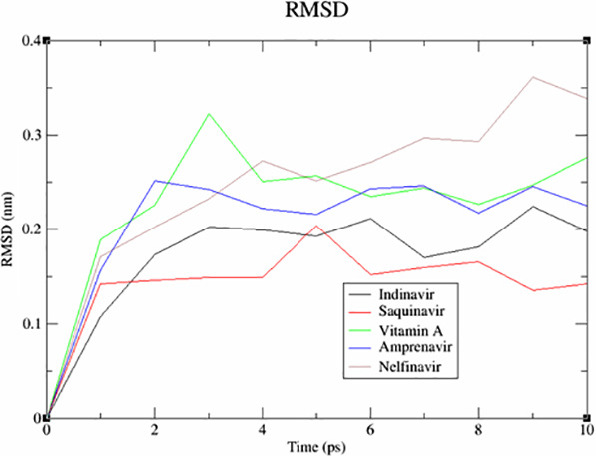
The analysis of RMSD for DOCK4.

**Figure 4:**
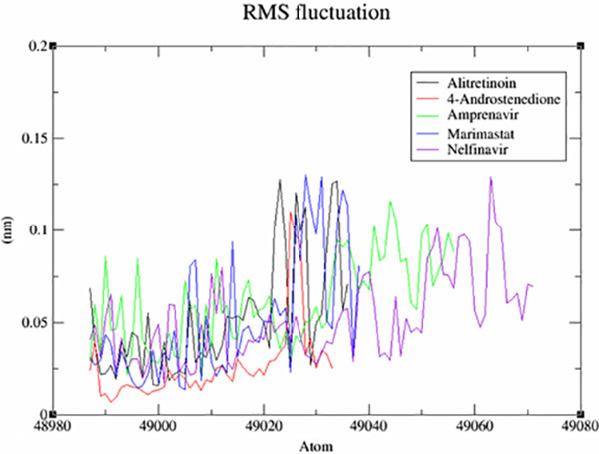
Comparative RMSF graph of system for NMDA.

**Figure 5:**
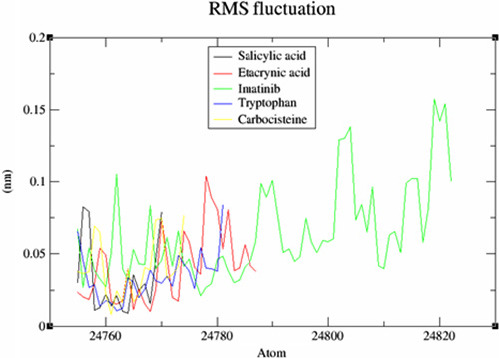
Comparative RMSF graph of system for AMPA.

**Figure 6:**
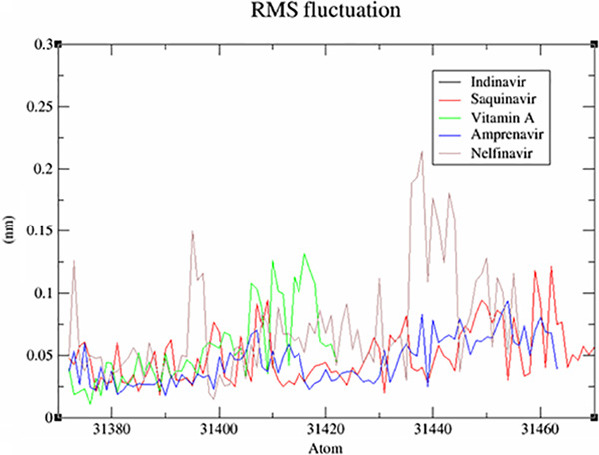
Comparative RMSF graph of system for DOCK4.

This screening involved a target protein (NMDA, AMPA and DOCK4) and 13 compounds utilised as ligands. The results can be observed in Figs. 1–6.

### In silico Toxicity Analysis (ADMET)

Table 2 illustrates the results of the ADMET toxicity prediction for the complete set of 13 drugs. The prediction methodology utilises a scope limitation, whereby only compounds that satisfy the condition of structural resemblance to the desired chemical are included in the predictive model. However, a thorough examination was undertaken to assess the unlimited applicability domain of the prediction model, and the results were presented clearly and separately. The depiction of the prediction results for the confined applicability domain utilised solid colours, while the representation of the prediction results for the unconstrained applicability domain employed no colours. The results derived from an unrestricted applicability domain generally exhibit a reduced level of dependability, as evidenced by a little decline in both accuracy and sensitivity, compared to the results obtained from a limited applicability domain (Schyman *et al*. 2017).

## DISCUSSION

### Acquisition and Verification of Protein Structure

The ERRAT algorithm investigates the non-bonded statistical interactions among different types of atoms. It represents the error function values about the location of a sliding window consisting of 9 residues. This positional information is obtained from meticulously refined structures (Dym *et al*. 2012). From Table 1 the ERRAT for NMDA is 74.4818, AMPA is 96.1992 and DOCK4 obtain 98.612.

The Verify-3D algorithm assesses the compatibility between a 3D model and its corresponding amino acid sequence by assigning a structural class based on environmental characteristics (such as loop, polar, non-polar, alpha, beta) and position. The algorithm then compares these results with other known structures (Pawlowski *et al*. 2008). The percentage of Verify-3D (Table 1) for NMDA is 60.33, AMPA is 23.95 and DOCK4 is 84.14.

The Ramachandran plot is utilised to assess the structural integrity of a given entity, primarily by evaluating the prevalence of regions deemed favourable or permissible. The results obtained from NMDA (7EOS) indicate a significant proportion of residues, precisely 95.8% (2937 out of 3066). AMPA (7F3O) exhibited a high level of conformational quality, with 99.1% (1573/1587) of its residues occupying preferred areas (98% threshold). The protein DOCK4 (Q8N1I0) exhibited a favourable conformation for 86.9% (1706/1964) of its total residues, corresponding to regions with a 98% likelihood. These results can be view in Table 1.

### Docking Results

AutoDock Vina is a widely used open-source programme for molecular docking. It is designed to perform docking simulations between small ligands and macromolecules. AutoDock Vina utilises a simple scoring function and rapid conformational search to calculate the best binding pose between the ligand and the target protein (Trott & Olson 2010). From Table 3, the drugs with the best binding energy for NMDA, AMPA and DOCK4 are marimastat, salicylic acid and vitamin A, have the best scores, respectively.

AutoDock4 is a suite of automated docking tools designed to predict how small molecules, such as substrates or drug candidates, bind to a protein. It allows for flexible modelling of both the ligand and specific portions of the protein, providing valuable insights into molecular interactions. (Morris *et al*. 2009). The drugs with the best binding energy for NMDA, AMPA and DOCK4 are alitretinoin, carbocisteine and amprenavir, which can be viewed in Table 4.

Then, Hdock is the following tool used for docking. Hdock is a web server that provides a highly integrated suite of protein-protein and protein-DNA docking tools. The server utilises a hybrid algorithm that combines template-based modelling and free docking to predict the interactions between proteins or protein-DNA complexes. It accepts sequence and structure inputs for proteins and supports various docking processes (Yan *et al*. 2017). From Table 5, NMDA has the best docking score with alitretinoin, which is −157.00. For AMPA, the best docking score is with salicylic acid, which is −94.47. Meanwhile, for DOCK4, the best docking score is for vitamin A, which is −137.97.

Next, CB docks were used for docking. CB-Dock is a software tool used in molecular docking for protein-ligand binding analysis and prediction. It is designed to improve the accuracy of protein-ligand blind docking by exploring the binding sites of receptors and identifying the corresponding binding poses of ligands (Liu *et al*. 2022). For NMDA, the best binding energy is marimastat. For AMPA, the best binding energy is with carbocisteine, and for DOCK4, the best binding energy is with both amprenavir and vitamin A (Table 6).

Based on all the results obtained from the four tools used, alitretinoin and marimastat have the best docking results when binding to NMDA, while salicylic acid and carbocisteine give the best docking results for AMPA. While for DOCK4, both amprenavir and vitamin A have the best docking score.

### Molecular Dynamic Using GROMACS

A molecular docking analysis was performed to evaluate which drugs have the best potential for restoring NMDA receptor function. Following this, a compound screening was conducted utilising MD simulations. The screening process encompassed the evaluation of three specific proteins (NMDA, AMPA and DOCK4) and the use of 13 drugs as ligands.

The influence of drug-protein interactions on the dynamics of biological systems is a fundamental facet of therapeutic development. This study utilised the root mean square deviation (RMSD) as a metric to investigate the impact of particular medications on the stability of NMDA, AMPA and DOCK4 proteins. The MD simulations, with a duration of 10 nanoseconds, were performed on a set of 15 drug-protein complexes using the GROMACS software. The measurements of the RMSD oscillations were conducted and presented afterwards. The RMSD was computed to assess the collective dynamics, stability and convergence of the various systems. The results are illustrated in Fig. 1 for NMDA, Fig. 2 for AMPA and Fig. 3 for DOCK4.

Based on the data presented in Fig. 1, it is evident that the binding of alitretinoin to NMDA leads to a significant decrease in the RMSD value. The black trace has a slight jump, so the ligand in the black trace which wobbles less might have an alternative binding mode. Not only that, lower deviation or less wobble correlates with more stable binding. Marimastat might have a steadier increase throughout. However, it has higher RMSD value which indicates there are bigger conformation changes in protein; hence the complexes are less stable. After doing a more thorough analysis, it was shown that the AMPA-salicylic acid (Fig. 2) was the highest. However, it has quite a high jump which indicates higher deviation and more wobble correlates with less stable binding. While carbocisteine has a steadier increase throughout. This might indicate more stable binding. In Fig. 3, the RMSD value of DOCK4-vitamin A has experienced a significant increase, possibly due to an alternative binding mode, as evidenced by the graph’s steady increase after the jump. While amprenavir has a steadier increase throughout, this might indicate a more stable binding.

A noteworthy characteristic of MD simulation relates to the flexibility of a protein’s backbone, which may be assessed by measuring the root-mean-square fluctuation (RMSF) metric. The results of the comparative study examining the effects of these drugs on NMDA, AMPA and DOCK4 receptors were graphically displayed in Figs. 4, 5 and 6, respectively. Upon conducting a more detailed analysis of Fig. 4, it becomes evident that including 4-Androstenedione led to a decrease in the oscillatory of the NMDA. The influence of tryptophan binding on the protein’s flexibility is seen in Fig. 5. The presence of tryptophan resulted in a reduction in the oscillations of the AMPA which might indicate that the complex structure is more rigid and well-defined compared to others. According to the data presented in Fig. 6, nelfinavir has the highest oscillations. These oscillations suggest that the loop regions possess higher conformational flexibility, leading to a less precisely defined structure.

### ADMET Studies

The findings of the ADMET toxicity prediction for all 13 drugs are depicted in Table 7. The prediction approach employs a scope constraint, wherein only compounds that meet the requirement of structural similarity to the requested chemical are incorporated into the prediction model. Nevertheless, an analysis was conducted on the prediction model’s unrestricted applicability domain, and the outcomes were displayed distinctly. Specifically, the restricted applicability domain prediction results were depicted using solid colours, whereas the unrestricted applicability domain prediction results were represented using striped colours. Typically, the outcome obtained from an unlimited applicability domain has a somewhat diminished level of reliability, characterised by a minor decrease in accuracy and sensitivity, compared to the outcome obtained from a restricted applicability domain (Schyman *et al*. 2017).

Alitretinoin was predicted to be positively toxic to DILI, H-HT and AMES tests and inhibit two out of five CYP enzymes (CYP2C9 and CYP2D6) besides being favourable to SR-MMP. While marimastat have a better result which only thought to be positive in H-HT test however it could not inhibit any Cyp inhibitors that being tested. Salicylic acid is predicted to be toxic and favourable to DILI and H-HT, and it can penetrate the blood-brain barrier (BBB). While carbocisteine on the other hand has negative results on all three toxicity tests tested however, it could neither pass through BBB nor inhibit any Cyp inhibitors. It was thought that vitamin A is less harmful to DILI, H-HT and AMES tests however it could not pass through BBB. While amprenavir has only negative results for the AMES test.

Alitretinoin and marimastat, salicylic acid and carbocisteine, and vitamin A and amprenavir interact most favourably with the NMDA, AMPA and DOCK4 receptors, in that order. However, the toxicity analysis shows a concerning result.

Even though the analysis was done using the in silico method and all the results seemed to be safe, there should be some precautions to proceed with these drugs for restoring the protein functions as the side effects and long-term effects might vary from the predicted results.

## CONCLUSION

This study found 13 Drug ReposER drugs that restored synaptic communication between NMDA, AMPA and DOCK4 protein. Docking tools included Autodock Vina, AutoDock4, Hdock and CB dock. Alitretinoin, salicylic acid and indinavir docked best to NMDA, AMPA and DOCK4.

GROMACS was used to simulate MD to study drug-protein binding. The all-atom force field CHARMM36 (July 2022) generated the topology file. Each graph’s trajectory was thoroughly reviewed to determine bond stability using the results. The RMSF oscillations of nelfinavir were among the highest, indicating that the loop sections had greater conformational flexibility and a less stable structure.

Following docking scores, ADMET predictors were utilised to assess each drug’s potential, particularly alitretinoin, salicylic acid and indinavir. This prediction included absorptive capacity, distribution in the body, metabolism, excretion and toxic effects. Only 4-androstenedione, tryptophan, carbocisteine and vitamin A were harmful to DILI, H-HT and AMES toxicity tests. These facts could minimise and eliminate any undesirable effects.

More studies need to be done to better understand potential side effects. This study could serve as a guide in constructing a framework for future studies.

## Figures and Tables

**Table 1:** Validation using SAVES v6.0.

Proteins	ERRAT	Verify-3D (%)	Z-score	Ramachandran score (%)
NMDA	74.4818	60.33	Pass	95.8
AMPA	96.1992	23.95	Pass	99.1
DOCK4	98.6120	84.14	Fail	86.9

**Table 2:** Result from Drug ReposER.

Proteins	Drugs
NMDA	Alitretinoin
	4-Androstenedione
	Amprenavir
	Marimastat
	Nelfinavir
AMPA	Salicylic acid
	Etacrynic acid
	Imatinib
	L-Tryptophan
	Carbocisteine
DOCK4	Indinavir
	Saquinavir
	Vitamin A
	Amprenavir
	Nelfinavir

**Table 3:** The analysis obtained from Autodock Vina software.

Proteins	Ligands (drug)	Binding energy (kcal/mol)
NMDA	Alitretinoin	−6.6
	4-Androstenedione	−7.8
	Amprenavir	−6.5
	Marimastat	−6.2
	Nelfinavir	−8.2
AMPA	Salicylic acid	−5.8
	Etacrynic acid	−6.6
	Imatinib	−9.3
	Tryptophan	−5.9
	Carbocisteine	−5.9
DOCK4	Indinavir	−8.1
	Saquinavir	−9.5
	Vitamin A	−5.9
	Amprenavir	−7.2
	Nelfinavir	−8.1

**Table 4:** The analysis obtained from AutoDock4 software.

Proteins	Ligands (drug)	Estimated free binding energy (kcal/mol)	Estimated inhibition constant, Ki (μM)
NMDA	Alitretinoin	−5.23	147.71
	4-Androstenedione	−6.88	9.11
	Amprenavir	−6.65	13.29
	Marimastat	−5.99	40.99
	Nelfinavir	−7.96	1.47
AMPA	Salicylic acid	−6.53	16.44
	Etacrynic acid	−6.62	13.95
	Imatinib	−5.92	45.77
	Tryptophan	−6.02	38.50
	Carbocisteine	−2.13	27.51 × 10^3^
DOCK4	Indinavir	−4.86	275.80
	Saquinavir	−7.64	2.51
	Vitamin A	−7.25	4.83
	Amprenavir	−3.63	2.18
	Nelfinavir	−4.15	903.76

**Table 5:** The results from Hdock.

Protein	Ligands (drugs)	Docking score	Structure quality
NMDA	Alitretinoin	−157.00	Low
	4-Androstenedione	−168.55	Good
	Amprenavir	−199.85	Good
	Marimastat	−168.23	Good
	Nelfinavir	−198.00	Good
AMPA	Salicylic acid	−94.47	Low
	Etacrynic acid	−122.86	Low
	Imatinib	−169.19	Low
	Tryptophan	−111.15	Low
	Carbocisteine	−99.77	Low
DOCK4	Indinavir	−204.32	Low
	Saquinavir	−204.27	Low
	Vitamin A	−137.97	Low
	Amprenavir	−199.88	Low
	Nelfinavir	−179.59	Low

**Table 6:** The results from CB-Dock.

Protein	Ligands (drugs)	Binding energy (kcal/mol)
NMDA	Alitretinoin	−8.0
	4-Androstenedione	−9.2
	Amprenavir	−8.4
	Marimastat	−6.0
	Nelfinavir	−9.7
AMPA	Salicylic acid	−5.8
	Etacrynic acid	−6.8
	Imatinib	−10.8
	Tryptophan	−6.9
	Carbocisteine	−4.6
DOCK4	Indinavir	−8.1
	Saquinavir	−8.8
	Vitamin A	−7.8
	Amprenavir	−7.8
	Nelfinavir	−9.0

**Table 7:** The 14 ADMET predictions for all 13 compounds are presented in a separate row. At least 3 compounds got more than 1 NPscore and 10 other compounds acquired less than 1 NPscore.

Drugs	Toxicity			Metabolism Cyp Inhibitors for					Membrane transporters				Others	
			
	DILI	H-HT	AMES	1A2	2C19	2C9	2D6	3A4	BBB	P-gp inhibitor	P-gp substrate	hERG Blocker	SR-MMP	NPscore
Alitretinoin	Yes	Yes	Yes	No	No	Yes	Yes	No	No	No	No	No	Yes	2.305
4-Androstenedione	No	No	No	No	No	No	No	No	Yes	Yes	No	No	Yes	2.424
Amprenavir	Yes	Yes	No	No	Yes	Yes	No	Yes	No	No	Yes	No	Yes	−0.198
Marimastat	No	Yes	No	No	No	No	No	No	Yes	No	Yes	No	No	0.438
Nelfinavir	Yes	Yes	No	No	Yes	Yes	Yes	Yes	No	Yes	No	No	Yes	−0.089
Salicylic acid	Yes	Yes	No	No	No	No	No	No	Yes	No	No	No	No	0.139
Etacrynic acid	Yes	Yes	No	Yes	No	Yes	No	No	No	No	No	No	No	0.105
Imatinib	Yes	Yes	No	No	Yes	No	No	Yes	Yes	Yes	Yes	Yes	Yes	−1.477
Tryptophan	No	No	No	No	No	No	No	No	Yes	Yes	Yes	No	No	0.392
Carbocisteine	No	No	No	No	No	No	No	No	No	No	No	No	No	0.306
Indinavir	No	Yes	No	No	No	No	Yes	Yes	Yes	Yes	No	Yes	Yes	−0.416
Saquinavir	Yes	Yes	No	No	No	Yes	No	Yes	No	Yes	Yes	No	Yes	−0.044
Vitamin A	No	No	No	Yes	Yes	No	Yes	Yes	No	No	No	No	No	2.615

*Notes*: The highlighted compounds are the compounds with the best results.

## APPENDICES

### Appendix A

#### The Autodock Vina was run using the following script

*#! /usr/bin/env python*
*# -*- coding: utf-8 -*-*
*#*
*# My first example with AutoDock Vina in python*
*#*

**from vina import** Vina

v = Vina(sf_name=’vina’)

v.set_receptor(‘*1iep_receptor*.pdbqt’)

v.set_ligand_from_file(‘1iep_ligand.pdbqt’)
v.compute_vina_maps(center=[15.190, 53.903, 16.917], box_size=[20, 20, 20])

*# Score the current pose*
energy = v.score()
print(‘Score before minimization: *%.3f* (kcal/mol)’ % energy[0])

*# Minimized locally the current pose*
energy_minimized = v.optimize()
print(‘Score after minimization : *%.3f* (kcal/mol)’ % energy_minimized[0])
v.write_pose(‘1iep_ligand_minimized.pdbqt’, overwrite=**True**)

*# Dock the ligand*
v.dock(exhaustiveness=32, n_poses=20)
v.write_poses(‘1iep_ligand_vina_out.pdbqt’, n_poses=5, overwrite=**True**)

### Appendix B

#### The Autodock was run using the following script

# Install openbabel, pymol, autodock
Sudo apt update

Sudo apt full-upgrade

Sudo apt install openbabel pymol

Pymol AutoDock.py -b FILENAME.pdb

Select an amino acid (must be amino acid only) where you want the center of the search
box to be then in PyMol command terminal Box (“sele”,1,1,1) where 1, 1, 1 are the x y z
distances from the center of the box

python3 AutoDock.py -r FILENAME.pdb

python3 AutoDock.py -d FILENAME.wget

python3 AutoDock.py -s FILENAME.pdbqt 300000

python3 AutoDock.py -j Center_X Center_Y Center_Z Size_X Size_Y Size_Z Seed

Exhaustiveness Output CPUs Array Email

python3 AutoDock.py -c DIRECTORY

python3 AutoDock.py -p pX pY pZ x y z seed exhaust out CPU array email

for file in ./Ligands/*/*; do tmp=${file%.pdbqt}; name=”${tmp##*/}”; ./vina --receptor
receptor.pdbqt --ligand “$file” --out $name_out.pdbqt --log $name.log --exhaustiveness
10 --center_x 0 --center_y 0 --center_z 10 --size_x 15 --size_y 15 --size_z 15; awk
‘/^[-+]+$/{getline;print FILENAME,$0}’ $name.log >> temp; done; sort temp -nk 3 >
Results; rm temp; mkdir logs; mv *.log *.out logs

### Appendix C

#### Command script for md simulation as follows

grep Ligand protein.pdb > ligand.pdb
grep -v HETATM protein.pdb > protein_protein.pdb
gmx pdb2gmx -f protein_processed.pdb -o protein_processed.gro -water spc

obabel -i sdf ligand.sdf -o mol2 -O ligand.mol2
perl sort_mol2_bonds.pl ligand.mol2 ligand_fix.mol2

python cgenff_charmm2gmx_py3_nx2.py Ligand ligand_fix.mol2 ligand.str charmm36-jul2022.
ff/

gmx editconf -f ligand_ini.pdb -o ligand.gro
gmx editconf -f complex.gro -o complex_box.gro -bt dodecahedron -d 1.0
gmx solvate -cp complex_box.gro -cs spc216.gro -p topol.top -o complex_solv.gro

gmx grompp -f ion.mdp -c complex_solv.gro -p topol.top -o ions.tpr
gmx genion -s ions.tpr -o complex_solv_ions.gro -p topol.top -pname NA -nname CL
-neutral
gmx grompp -f em.mdp -c complex_solv_ions.gro -p topol.top -o em.tpr
gmx mdrun -v -deffnm em

gmx make_ndx -f ligand.gro -o index_ligand.ndx
gmx genrestr -f ligand.gro -n index_ligand.ndx -o posre_ligand.itp
gmx make_ndx -f em.gro -o index.ndx

gmx grompp -f nvt.mdp -c em.gro -r em.gro -p topol.top -n index.ndx -o nvt.tpr
gmx mdrun -v -deffnm nvt

gmx energy -f em.edr -o potential.xvg

gmx energy -f nvt.edr -o temperature.xvg

gmx grompp -f npt.mdp -c nvt.gro -t nvt.cpt -r nvt.gro -p topol.top -n index.ndx -o
npt.tpr
gmx mdrun -v -deffnm npt

gmx energy -f npt.edr -o pressure.xvg

gmx energy -f npt.edr -o density.xvg

gmx grompp -f md.mdp -c npt.gro -t npt.cpt -p topol.top -n index.ndx -o md.tpr
gmx mdrun -v -deffnm md

gmx trjconv -s MD.tpr -f MD.xtc -o MD_center.xtc -center -pbc mol -ur compact
gmx trjconv -s MD.tpr -f MD_center.xtc -o start.pdb -dump 0
gmx rms -s MD.tpr -f MD_center.xtc -o rmsd.xvg
xmgrace rmsd.xvg

gmx rmsf -s MD.tpr -f MD_center.xtc -o rmsf.xvg
xmgrace output.xvg
